# Screening and Expression Characteristics of Trehalose Transporter Genes in the Parasitic Wasp *Baryscapus dioryctriae* (Hymenoptera: Eulophidae)

**DOI:** 10.3390/insects17101011

**Published:** 2026-09-30

**Authors:** Jian Zhou, Jing Li, Yinuo Li, Qiuyao Li, Feiyue Ding, Shaohan Huang, Bingzhong Ren, Qi Chen

**Affiliations:** 1 Jilin Provincial Key Laboratory of Animal Resource Conservation and Utilization, School of Life Sciences, Northeast Normal University, Changchun 130024, China; zhouj321@nenu.edu.cn (J.Z.); liyinuo596@nenu.edu.cn (Y.L.); liqiuyao@nenu.edu.cn (Q.L.); fyding657@nenu.edu.cn (F.D.); huangshaohan656@nenu.edu.cn (S.H.); bzren@nenu.edu.cn (B.R.); 2 Key Laboratory of Vegetation Ecology, Ministry of Education, Northeast Normal University, Changchun 130024, China; 3 Research Institute of Forest Protection, Jilin Provincial Academy of Forestry Sciences, Changchun 130033, China; lij9030@163.com; 4 Jilin Province Main Forest Diseases and Insect Pests Monitoring, Epidemic Prevention Technology Innovation Center, Changchun 130033, China; 5 Jilin Provincial International Cooperation Key Laboratory for Biological Control of Agricultural Pests, Changchun 130118, China

**Keywords:** *Baryscapus dioryctriae*, gene family expansion, trehalose transporters, RT-qPCR, spatiotemporal expression

## Abstract

Trehalose serves as both a hemolymph sugar and stress protectant, playing pivotal roles in insect energy metabolism, development, and stress adaptation. Its transmembrane transport is mediated by trehalose transporters (TRETs). *Baryscapus dioryctriae*, a parasitoid wasp targeting the red pine shoot borer, shows great potential for biocontrol. Prior genomic assemblies indicated prominent expansion of the *Tret* gene family in this wasp, yet systematic characterization of these genes has not been reported. Here, leveraging a chromosome-level genome, we identified 94 *BdioTrets*. Phylogenetic analysis placed them closest to *Phytomastichus coffea Trets* (maximum identity: 88.15%). Most members conserved the GRR/K motif, N-glycosylation sites, and key residues in TM7/TM10. Fourteen candidate genes were further verified via reverse transcription quantitative PCR (RT-qPCR), and Pearson correlation analysis showed that 11 of the 14 candidate genes have strong and significant positive correlations for their tissue expression profiles (*r* > 0.7, *p* < 0.05), indicating consistent expression trends between RNA-seq and RT-qPCR. These genes exhibited stage-specific expression: *BdioTret9* peaked in eggs; *BdioTret3*,*5* and *67* in larvae; *BdioTret25* in prepupae; and *BdioTret6* in pupae, while eight others dominated in adults. Tissue-specific profiling revealed pronounced sexual dimorphism in antennal expression and distinct localization in the abdomen (sex-biased) and legs. Our findings demonstrate that the large-scale *BdioTrets* expansion, coupled with precise spatiotemporal regulation, establishes a flexible energy network supporting the parasitic lifecycle. These results lay a fundamental framework for future functional investigations of TRETs in hymenopteran parasitoids.

## Simple Summary

Trehalose is the principal circulating sugar in insect hemolymph for insects and requires trehalose transporters (TRETs) to cross cell membranes. In this study, we analyzed the genome of *Baryscapus dioryctriae*, a parasitic wasp used for forest pest control and identified 94 *Tret* genes—the largest TRET family reported to date in Hymenoptera. These genes exhibit distinct expression patterns across all life stages (egg, larva, pupa, and adult) and in various tissues, including antennae, abdomens, and legs. Notably, we observed significant expression differences between male and female antennas. The massive expansion and precise spatiotemporal regulation of these *Tret* genes establish a flexible energy network that supports the wasp’s parasitic lifestyle. These findings provide a theoretical basis for the selective breeding of this wasp and its application in biological pest control.

## 1. Introduction

Trehalose is a non-reducing disaccharide formed by two glucose molecules linked via an α, α-1,1-glycosidic linkage. It broadly exists in bacteria, fungi, and invertebrates as well as plants, yet is absent in vertebrates [1]. Beyond serving as a primary energy reserve, trehalose functions as a chemical chaperone to mitigate abiotic stresses, regulates protein homeostasis, and acts as a signaling molecule in plants [2,3,4,5,6,7,8,9]. Trehalose cannot diffuse passively through cell membranes; its transmembrane translocation therefore solely relies on trehalose transporters (TRETs) [10]. TRETs belong to the Solute Carrier Family 2 (SLC2A) within the Major Facilitator Superfamily (MFS), and are classified into three subtypes: TRET1, TRET1-2 and TRET1-like, typically containing 10–12 transmembrane α-helices [11,12]. Systematic phylogenetic analyses demonstrate that insect TRET1 homologs form a distinct monophyletic clade, clearly separated from the human GLUT/SLC2A family and the Drosophila melanogasterGLUT1 ortholog, DmGLUT1 (CG1086-PB) [13], indicating that TRET1, while sharing a common ancestor with the GLUT/SLC2A family, has undergone significant functional divergence to specifically mediate trehalose transport. They drive trehalose translocation via facilitated diffusion or ion-coupled active transport, and conserve core functional motifs including the GRR/K residue, N-glycosylation sites, and substrate-recognition residues in transmembrane domains [13]. Accumulating evidence demonstrates that TRETs underpin insect desiccation tolerance, developmental progression, stress resistance and immune responses [1,14,15,16,17,18], establishing their irreplaceable role in insect physiology.

Gene family expansion acts as a major evolutionary driver facilitating insect environmental adaptation, producing duplicated paralogs that diverge through subfunctionalization or neofunctionalization [19]. Well-studied expanded gene families include cytochrome P450 monooxygenases (P450s) conferring enhanced detoxification against plant allelochemicals and synthetic insecticides [20,21,22,23]; phosphofructokinase (PFK), whose duplication boosts glycolysis to satisfy energy demands under stress [24]; and chemosensory receptor genes (ORs, GRs, IRs) with lineage-specific expansions facilitating host localization and foraging [25,26,27]. The trehalose transporter family also undergoes frequent expansion in insects, particularly in Lepidoptera: 69 *Trets* are identified in *Helicoverpa armigera*, 79–89 in *Spodoptera frugiperda*, and 88 in *Bombyx mori*, with duplicated members contributing to metabolic homeostasis and stress adaptation [11,19,28]. Functional validations in these species confirm that *Tret* expansion underlies fitness advantages under fluctuating environmental conditions.

*Baryscapus dioryctriae* (Hymenoptera: Eulophidae) is a pupal endoparasitoid wasp that primarily targets *Dioryctria abietella* and *Dioryctria rubella*, with broad potential for biocontrol of forestry and agricultural pyralid pests in Northeast China. Our teams assembled a chromosome-level genome of *B. dioryctriae* (485.5 Mb, contig N50 = 2.17 Mb, 6 fully anchored chromosomes) [29], revealing significant expansion of olfactory-related gene families. Functional characterization of the expanded *BdioOBP45*, which is specifically expressed in the ovipositor and binds γ-terpinene to mediate host location, highlights the ecological relevance of gene family expansion in this parasitoid. Preliminary genomic analyses by our group further identified marked expansion of the *BdioTret* family in *B. dioryctriae* [29]. Nevertheless, genome-wide identification, spatiotemporal expression profiling, and functional characterization of this expanded BdioTret family have not been systematically performed, restricting our understanding of their physiological roles in parasitoid biology.

In this study, we systematically characterized all *BdioTret* members based on the published chromosome-level genome of *B. dioryctriae* to systematically identify *Tret* family members, and characterize their phylogenetic relationships, conserved structural features and spatiotemporal expression patterns via integrated bioinformatics analysis, transcriptomic profiling and RT-qPCR validation. Our findings provide foundational data for dissecting the functional divergence of TRETs in hymenopteran parasitoids and offer theoretical guidance for elite germplasm screening and optimized artificial rearing of *B. dioryctriae* for biocontrol applications.

## 2. Methods

### 2.1. Insect Rearing

The parasitic wasps *B. dioryctriae* were originally collected from the Research Institute of Forest Protection, Jilin Provincial Academy of Forestry Sciences, and subsequently maintained at the School of Life Sciences, Northeast Normal University (Changchun, China). The parasitoids were reared in a controlled climate chamber (Model DNP-9082A, Xiyuan Science Instrument Co., Ltd., Shanghai, China) at 25 ± 1 °C, 55 ± 5% relative humidity (RH), with a 16 L: 8 D photoperiod, using *G. mellonella* as the sole host. Newly emerged adults were kept under identical conditions and supplied with 10% (*v*/*v*) sucrose solution as food.

### 2.2. Identification and Phylogenetic Analysis of BdioTrets

Based on previously generated genomic data for *B. dioryctriae* available in our laboratory (PRJNA1099397) [29], candidate genes were initially screened from the genome annotation files using the keywords “tret”, “trehalose”, “transporter”, and “trehalose transporter”. The corresponding gene IDs were retrieved, and their nucleotide sequences were extracted accordingly. All 94 *BdioTret* sequences identified in this study are annotated within the chromosome-level genome assembly of *B. dioryctriae* (GenBank assembly accession: GCA_040869075.1). Open reading frames (ORFs) and deduced amino acid sequences were predicted via the Getorf tool (ORF Extraction and Statistics v2.0) on the BMKCloud online platform (Beijing BMK Cloud Technology Co., Ltd., Beijing, China, https://international.biocloud.net, accessed on 12 March 2026). Subsequently, the deduced amino acid sequences were subjected to Blastp searches against the NCBI non-redundant protein database (https://blast.ncbi.nlm.nih.gov/Blast.cgi/, accessed on 8 April 2026) to confirm sequence homology. Reciprocal Blast searches were further applied to validate candidate *BdioTret* genes obtained from keyword screening and Blast. The presence of conserved functional motifs was verified using the NCBI Conserved Domain Database (CDD, v3.21) (http://www.ncbi.nlm.nih.gov/cdd/, accessed on 25 March 2026). Transmembrane helices were predicted using the TMHMM 2.0 server (https://services.healthtech.dtu.dk/services/TMHMM-2.0/, accessed on 20 April 2026). Finally, a comprehensive evaluation was conducted to ascertain if the identified genes constituted full-length coding sequences. This assessment hinged on three factors: ORF integrity (defined by a complete start/stop codon pair and absence of internal premature termination codons), fragment length validation via alignment with known homologs to rule out truncation, and the predicted number of transmembrane domains (typically 10–12 for functional MFS transporters).

On the basis of functional studies of Trets in Hymenoptera and Blastp results from model species, seven TRET protein sequences from representative hymenopteran species were selected for phylogenetic analysis (Table S2). The selected species included *Aphidius gifuensis*, *Apis mellifera*, *Bombus impatiens*, *Copidosoma floridanum*, *Trichogramma dendrolimi*, *Trichogramma pretiosum*, and *Phytomastichus coffea*. All protein sequences were retrieved from the National Center for Biotechnology Information (NCBI) database. Amino acid sequences were multiple-aligned using MAFFT v7. Poorly aligned positions were removed with TrimAl v1.4 using the automated1 mode. The optimal amino acid substitution model was selected by ModelFinder integrated in TBtools-II. ModelFinder implemented in TBtools was used to select the optimal amino acid substitution model. Based on the Bayesian Information Criterion (BIC), the LG + F + R7 model was identified as the best-fitting model. The maximum-likelihood (ML) phylogenetic tree was constructed with 5000 bootstrap replicates to evaluate node support. The ML tree was mid-point rooted. Partial and truncated sequences were retained in the final alignment and annotated on the phylogenetic tree. FigTree v1.4.4 was used for tree visualization and manual inspection.

Fragments Per Kilobase of transcript per Million mapped reads (FPKM) values corresponding to the identified *BdioTrets* were retrieved from the laboratory’s existing transcriptomic dataset of *B. dioryctriae* using the respective gene IDs (SRR21665100-SRR21665109). The expression data were transformed using log_2_ (FPKM + 1) and imported into the Hiplot online platform (https://hiplot.com.cn/cloud-tool/self-operated/detail/106, accessed on 12 August 2026) to generate a heatmap. Clustering was performed using the Ward.D2 method with Euclidean distance as the metric.

The screening of candidate *BdioTret* genes was performed as a descriptive and exploratory analysis to prioritize genes for downstream validation. Following methodologies in insect analyses [30,31], a data-driven percentile-based threshold (the 95th percentile of log_2_(FPKM + 1) values across all 94 *BdioTret* genes and all examined tissues) was employed to preselect candidate genes from the initial set. Descriptive statistics and percentile values were calculated using SPSS Statistics 27. Genes with log_2_(FPKM + 1) values exceeding this threshold in at least one tissue were considered to exhibit relatively high transcript abundance among all 94 *BdioTret* genes. Candidate *BdioTrets* were preliminarily determined by integrating the gene-specific expression profiles, the topology of the phylogenetic tree, and previously reported characteristics of expanded BdioTRETs.

Candidate *BdioTrets* sequences obtained from the above pipeline were classified into three subtypes based on the following criteria: Specifically, *Tret1* genes clustered with known TRET1 orthologs (e.g., DmTRET1-1, PcofTRET1) in the ML phylogenetic tree constructed from seven hymenopteran species, possessed a complete ORF and 12 transmembrane domains, and retained the core TRET1 motifs (GRR/K, N-glycosylation site, TM7 QL/FS, and TM10 W). *Tret1-2* genes clustered with known TRET1-2 orthologs (e.g., DmTRET1-2, PcofTRET1-2) in the phylogenetic tree, exhibited sequence features characteristic of the TRET1-2 subtype, and typically lacked the N-terminal intracellular region. Tret1-like genes also possessed the MFS conserved domain and 10–12 transmembrane domains but formed independent sub-branches distinct from *Tret1*/*Tret1-2* in the phylogenetic tree, with lower retention of core motifs.

Subsequently, the amino acid sequence of AmTRET1 (NP_001107211.1) from the honey bee *Apis mellifera*, a representative insect in Hymenoptera, and DmTRET1 (NP_725068.1) from the well-characterized model organism *Drosophila melanogaster* were selected as the reference sequences. The AmTRET1 protein structure (UniProt: A9ZSY2), with three-dimensional coordinates retrieved from the AlphaFold database (RCSB AF_AFA9ZSY2F1), was then used as the structural template. Candidate TRET protein sequences were aligned using MEGA11. Multiple sequence alignment was performed via the Clustalw server (https://www.genome.jp/tools-bin/clustalw, accessed on 12 August 2026). The alignment results were visualized and analyzed using ESPript 3.0 (https://espript.ibcp.fr/ESPript/ESPript/, accessed on 12 August 2026) and combined with conserved domain predictions to further characterize the structural features of the candidate sequences.

### 2.3. Sample Collection, RNA Extraction and cDNA Synthesis

#### 2.3.1. Sample Collection and Processing

A series of samples were subjected to RT-qPCR analysis, including *B. dioryctriae* eggs dissected from parasitized *G. mellonella* pupae, as well as multiple developmental-stage samples and diverse adult tissues of *B. dioryctriae*. Pupae of *G. mellonella* were transferred into a Petri dish filled with 2 mL PBS buffer. Sterile forceps disinfected with absolute ethanol and RNase-free water were used to dissect pupae, and eggs collected at 36 h post oviposition were isolated with a micropipette under an optical microscope (Olympus BX51T, Olympus Corporation, Tokyo, Japan). Larval, prepupal, pupal and adult individuals of *B. dioryctriae* were manually screened under microscopy (Nikon SMZ1500, Nikon Corporation, Tokyo, Japan). Prior to tissue dissection, adult wasps were anesthetized on ice for 15 min. Dissected tissues included male and female antennae, male and female genitalia, male and female abdomens (digestive tracts and gonads removed), mixed-sex thoraxes, and mixed-sex legs. The numbers of insects pooled per tissue for each individual biological replicate are as follows: 200 male and female adult antennae, 200 female ovipositors, 200 male genitalia, 100 male and female adult abdomens (digestive tract and gonads removed), 100 thoraxes (mixed sexes), and 100 legs (mixed sexes) of *B. dioryctriae* adults. Additionally, for samples from the eggs (eggs dissected from parasitized *G. mellonella* pupae), larval, prepupal, pupal, and adult stages of *B. dioryctriae*, the numbers of insects pooled per biological replicate were 1000 eggs, 100 larvae, 100 prepupae, and 100 pupae, with 80 adults. All freshly harvested samples were immediately transferred into 1.5 mL RNase-free centrifuge tubes pre-filled with 1 mL 75% ethanol (diluted with DEPC-treated water). The samples were centrifuged at 14,000 rpm for 5 min at 4 °C. The supernatant was gently discarded, and the resulting pellets were snap-frozen in liquid nitrogen for a minimum of 30 min before long-term storage at −80 °C for downstream experiments.

#### 2.3.2. Total RNA Extraction and cDNA Synthesis

Total RNA was extracted using the HiPure Universal RNA Mini Kit (Magen Biotechnology Co., Ltd., Guangzhou, China) according to the manufacturer’s instructions. Following extraction, RNA purity and concentration were assessed using a Nanodrop 2000 micro-spectrophotometer (Thermo Fisher Scientific, Waltham, MA, USA). The remaining RNA samples were stored at –80 °C until further use. First-strand cDNA synthesis was performed using the One-Step gDNA Removal and cDNA Synthesis SuperMix (TransGen Biotech, Beijing, China) according to the manufacturer’s protocol. The thermal cycling program was configured as follows: 42 °C for 30 min, followed by 85 °C for 5 s to inactivate the reverse transcriptase. The resulting cDNA products were aliquoted into 5 µL volumes per tube and stored at –20 °C. Prior to downstream applications, the cDNA was diluted at a ratio of 1:5 with nuclease-free water.

### 2.4. Reverse Transcription Quantitative PCR Analysis

Based on the verification by BestKeeper [32] and our previous study [29], the Ribosomal protein L18 gene (*BdioRPL18*) and Glyceraldehyde-3-phosphate dehydrogenase gene (*BdioGAPDH*) were selected as reference genes. BestKeeper analysis showed that both *BdioRPL18* (SD = 0.754; CV = 3.40%) and *BdioGAPDH* (SD = 0.965; CV = 3.86%) met the stability criterion [32]. Pearson correlation analysis revealed no significant co-expression between the two genes (*r* = 0.089, *p* = 0.773), and both showed significant positive correlations with the BestKeeper index (*BdioRPL18*: *r* = 0.667, *p* = 0.013; *BdioGAPDH*: *r* = 0.801, *p* = 0.001). Therefore, both genes were selected as reference genes. The standard curves for primer efficiency were generated using ten-fold serial dilutions of pooled cDNA prepared by mixing equal amounts of eight individual cDNA templates [33]. Both *BdioRPL18* and *BdioGAPDH* exhibited high linearity (R^2^ > 0.99) and acceptable amplification efficiencies within the range of 90–110%, complying with the MIQE guidelines [34,35]. The corresponding standard curves are shown in Figure S1. Specific primers for the reference genes and *BdioTrets* were designed using Primer Premier 5 software (Table S1). Amplicon lengths were designed to be between 80 and 150 bp, and primer specificity was verified using TBtools software. RT-qPCR assays were performed using PerfectStart^®^ Green qPCR SuperMix (TransGen Biotech, Beijing, China) according to the manufacturer’s instructions. The reactions were run on a LightCycler 480 II Detection System (Roche, Shanghai, China) with the following thermal profile: initial denaturation at 94 °C for 30 s; followed by 45 cycles of 94 °C for 5 s, 55 °C for 15 s, and 72 °C for 10 s. Melting curve analysis was conducted under the following conditions: 95 °C for 5 s, 65 °C for 1 min, 97 °C for 10 s, and 60 °C for 15 s. All RT-qPCR assays were performed in triplicate (three technical replicates per sample) with three biological replicates. Two negative controls (no target and no reverse transcriptase) were included for each gene. Relative transcript abundance was quantified based on the 2^−ΔΔCt^ method [36]. Statistical analyses were conducted via one-way ANOVA followed by Tukey’s HSD multiple comparison test in GraphPad Prism v10.4.1. All data are displayed as mean ± standard deviation (SD), and distinct lowercase letters above bars denote significant intergroup differences (*p* < 0.05).

## 3. Results

### 3.1. Identification of Trehalose Transporter Genes in B. dioryctriae

The results obtained by reciprocal Blast are consistent with those of Blastp. A total of 94 *BdioTrets* were identified from the genomic data of *B. dioryctriae*. Among the 94 identified *BdioTrets* genes, the deduced amino acid lengths of the encoded proteins ranged from 163 to 1251 residues, including 77 full-length sequences with 10–12 TMDs, one long sequence, *BdioTret1* (1251 amino acids with 15 predicted TMDs), four sequences (*BdioTret42*, *54*, *55*, and *80*) with fewer than 10 predicted TMDs, along with 12 short truncated sequences (*BdioTret83*–*94*). And comprising five *BdioTret1* members (*BdioTret3*, *6*, *44*, *56*, and *79*), four *BdioTret1–2* members (*BdioTret2*, *4*, *36*, and *82*), and 85 *BdioTret1-like* members (Table 1). The genes were systematically renamed using Roman numerals I to XCIV based on the length of their deduced amino acid sequences. The longest protein encoded 1251 amino acids, while the shortest encoded 163. Most of the proteins (81 out of 94, 86.17%) were between 400 and 600 amino acids in length, with 11 proteins falling within the 290–400 amino acid range. Blastp analysis revealed that the *BdioTrets* primarily matched *PcofTrets*, with the highest sequence identity reaching 88.15%.

### 3.2. Phylogenetic Analysis of BdioTrets

**Figure 1:**
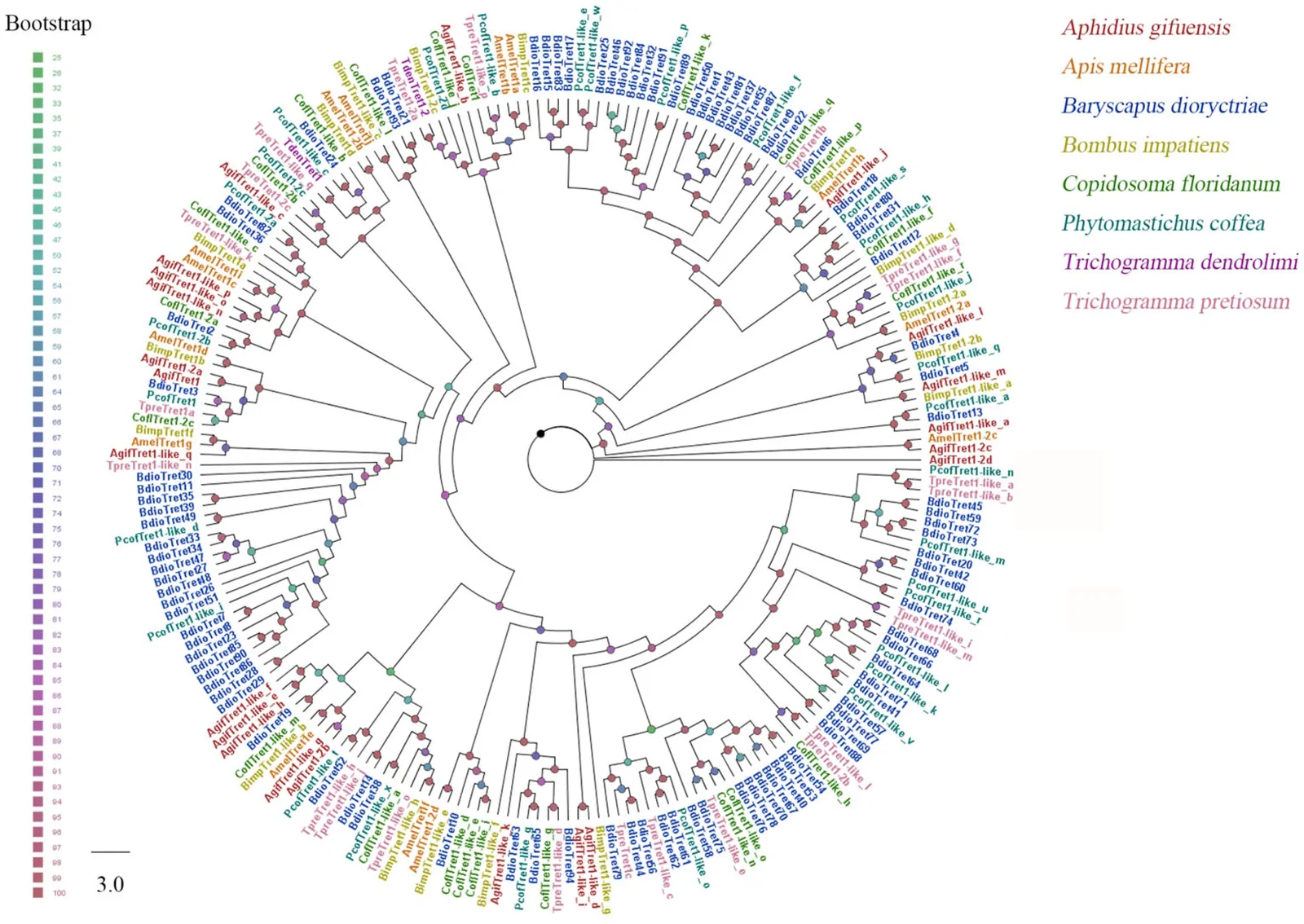
Phylogenetic tree of TRETs from seven Hymenoptera insects. *Agif*, *Aphidius gifuensis*; *Amel*, *Apis mellifera*; *Bimp*, *Bombus impatiens*; *Cofl*, *Copidosoma floridanum*; *Pcof*, *Phytomastichus coffea*; *Tden*, *Trichogramma dendrolimi*; *Tpre*, *Trichogramma pretiosum*.

Based on the ML phylogenetic tree of seven hymenopteran TRETs (Figure 1), combined with Blastp best-hit results (Table 1) and conserved motif analysis, the 94 BdioTRETs could be clearly classified into three subtypes. Specifically, five BdioTRETs (*BdioTret3*, *6*, *44*, *56*, and *79*) clustered with PcofTRET1 (XP_058798228.1) and their Blastp best hits were all annotated as “facilitated trehalose transporter *Tret1*”; thus, they were classified as *Tret1*. Four BdioTRETs (*BdioTret2*, *4*, *36*, and *82*) clustered with PcofTRET1-2 and their Blastp best hits were annotated as “facilitated trehalose transporter *Tret1-2* homolog”; thus, they were classified as *Tret1-2*. The remaining 85 BdioTRETs all possessed the MFS conserved domain but were distributed across multiple independent sub-branches within the *Tret1* backbone in the phylogenetic tree, and were therefore classified as *Tret1-like*. Notably, 73.4% of BdioTRETs clustered within a single clade alongside PcofTRETs, a pattern corroborated by the Blastp analysis.

### 3.3. Expression Profile Analysis of BdioTrets

**Figure 2:**
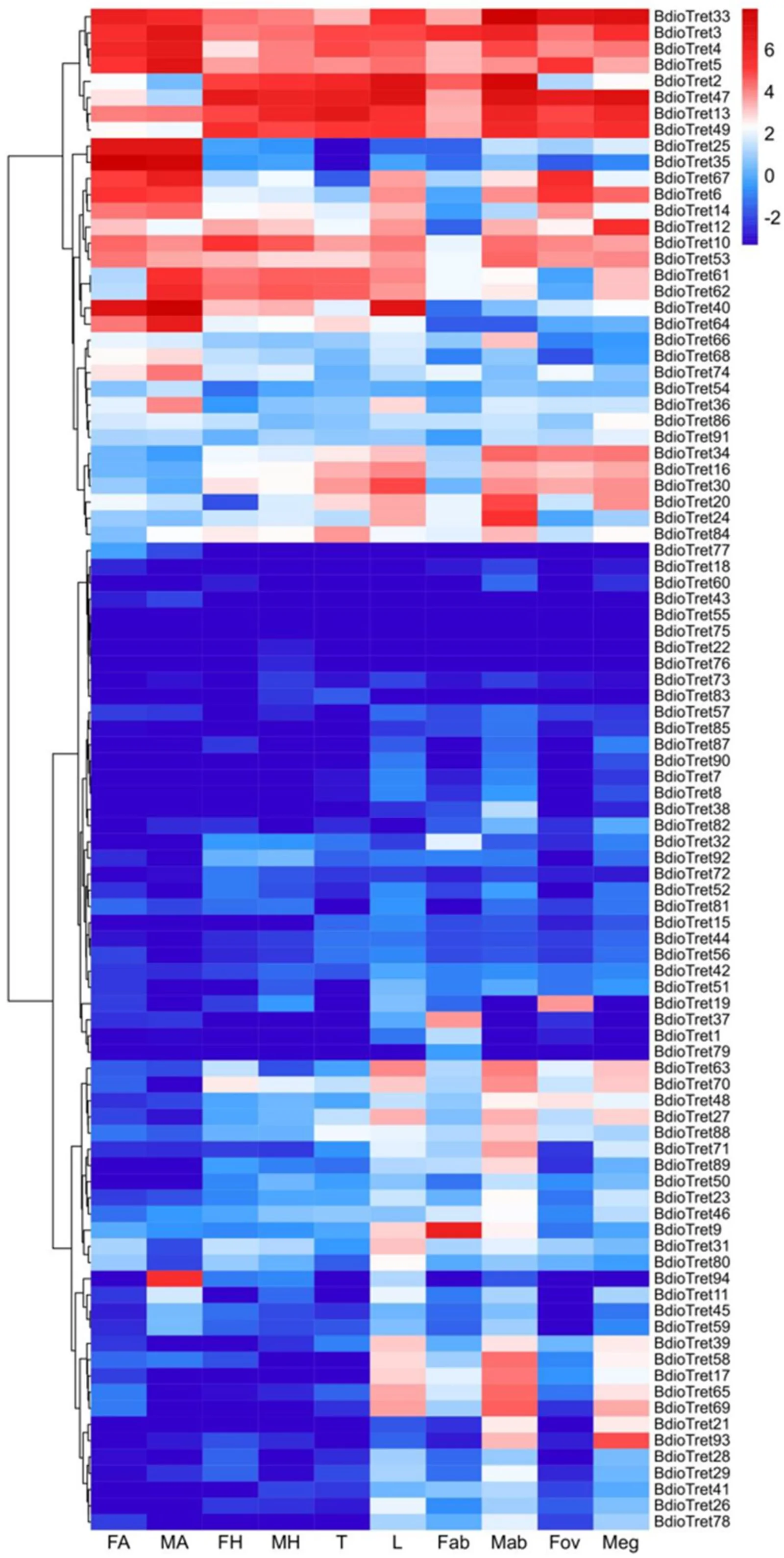
Heatmap of the expression levels of 94 *BdioTrets.* FA, female antennae; MA, male antennae; FH, female heads (without antennae); MH, male heads (without antennae); T, male and female thoraxes; L, male and female legs; Fab, female abdomens without ovipositors and digestive tracts; Mab, male abdomens without genitalia and digestive tracts; Fov, female ovipositors; Meg, male genitalia. Note: Raw expression values were log_2_(FPKM + 1), and row-wise z-score normalization was applied for heatmap visualization. The color bar represents z-score values ranging from -2 (dark blue) to 6 (dark red).

The expression heatmap of all 94 *BdioTrets* revealed distinct expression patterns based on relative transcript abundance derived from log_2_(FPKM + 1) values (Figure 2). Genes with log_2_(FPKM + 1) ≥ 5.23 (the global 95th percentile of all *BdioTret* genes–tissue expression values) were described as having relatively high transcript abundance, while genes with log_2_(FPKM + 1) < 5.23 were described as having relatively low transcript abundance. This threshold was determined descriptively as part of an exploratory screening strategy. The full descriptive statistics and percentile values are provided in Supplementary Table S3. Among all the identified 94 *BdioTrets*, roughly half of the genes exhibited very low or nearly undetectable transcript abundance, while the remaining genes displayed apparent tissue-biased expression profiles. According to these tissue-related abundance patterns, all *BdioTret* genes were grouped into three clusters and comparisons were made relative to each gene’s own signal across the sampled tissues. Cluster I contained genes with relatively high transcript abundance in antennae and showed no differential expression between females and males: *BdioTret25* and *BdioTret35* showed predominant transcript accumulation in antennae; *BdioTret6*, *BdioTret14* and *BdioTret67* showed high transcript abundance in both antennae and ovipositors; and *BdioTret40* and *BdioTret64* revealed relatively high transcript abundance in antennae and legs (pooled samples of both sexes). Cluster II comprised abdomen-biased genes: *BdioTret9* and *BdioTret37* showed elevated transcript abundance in female abdomens, whereas *BdioTret20*, *BdioTret24*, *BdioTret65* and *BdioTret69* presented comparatively higher transcript abundance than other *BdioTrets* in male abdomens and male genitalia. Cluster III included genes with broadly high transcript abundance across tissues: *BdioTret3*, *BdioTret4*, *BdioTret5* and *BdioTret13* maintained relatively high transcript abundance in all examined tissues, and *BdioTret47* and *BdioTret49* showed elevated transcript abundance in all tissues except antennae. In addition, *BdioTrets* exhibited highly similar expression patterns in certain tissues, such as female antennae versus male antennae; male abdomen and male genitalia; female antenna-free heads and male antenna-free heads; as well as mixed-sex samples for thoraxes, and legs (pooled samples of both sexes).

### 3.4. Motif and Conserved Domain Analysis of BdioTRETs

**Figure 3:**
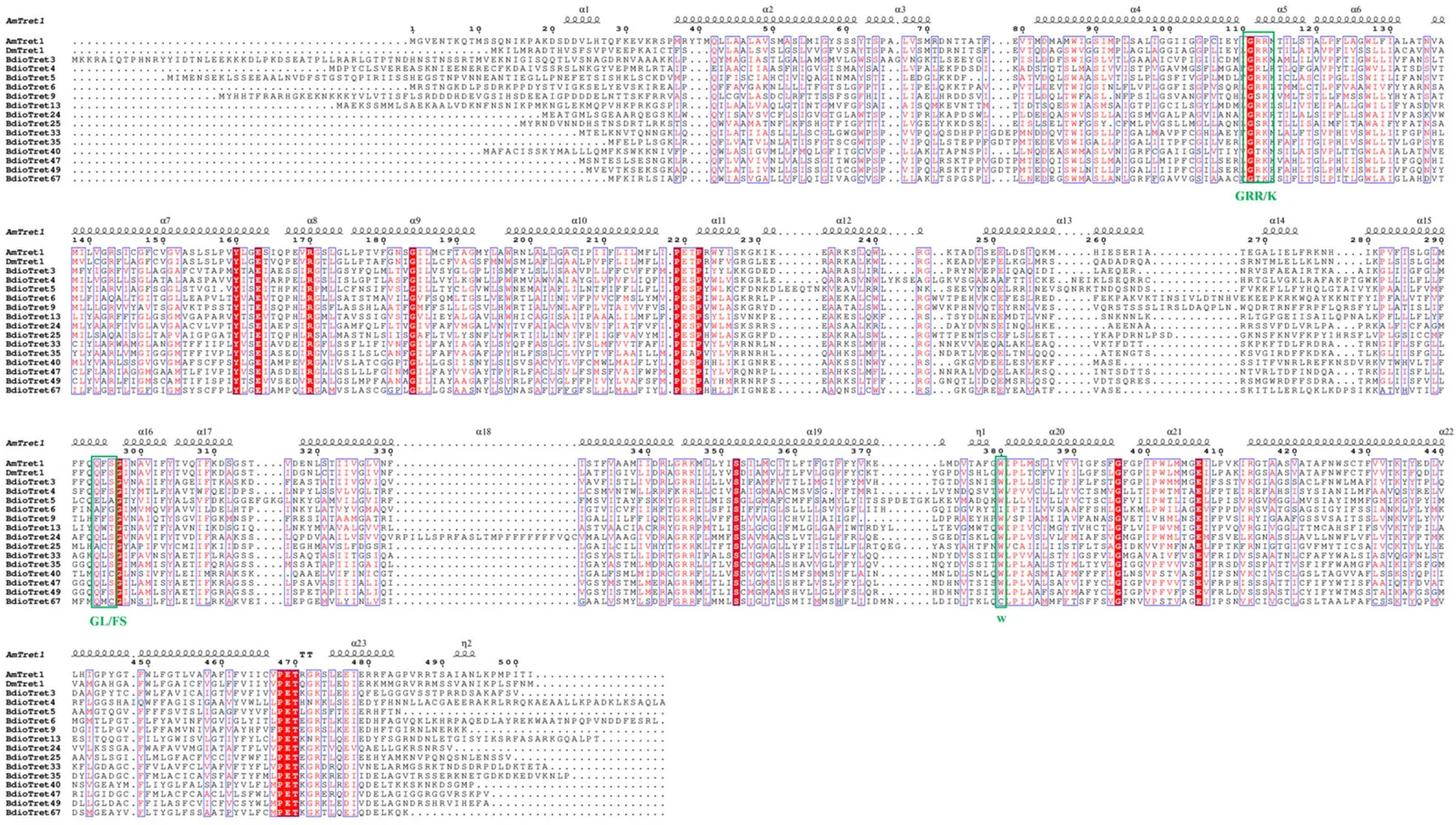
Sequence alignment of 14 candidate BdioTRETs with AmTRET1 and DmTRET1.

Using the structurally validated Hymenopteran AmTRET1 and Dipteran DmTRET1 as references, multiple sequence alignment revealed that the candidate BdioTRETs universally retain key functional motifs. Conserved domain prediction for the 94 identified *BdioTrets* yielded two distinct classifications (Figure 3): one comprising the Major Facilitator Superfamily (MFS) domain alone, and the other containing both the MFS domain and the MFS_GLUT6_8_Class3_like domain. All identified proteins conformed to the established criteria for Tret family members. Transmembrane-topology analysis indicated that among the 82 identified full-length *BdioTrets* sequences, sequences with 10–12 predicted transmembrane domains (TMDs) accounted for 93.90% of all full-length sequences. Of these, 50 sequences contained 12 TMDs, representing 60.98% of the total full-length sequences. This observation is consistent with the canonical structural features of functional TRETs. Building on the comparative genomic study by our team [29], a subset of *BdioTrets* was identified as having undergone significant expansion in *B. dioryctriae*. This expanded cohort includes *BdioTret20*, *40–42*, *44*, *45*, *53*, *54*, *56–62*, *64–66*, *68*, *70–75*, and *77*. The expansion of these specific genes suggests that they likely play pivotal roles in the ecological adaptation and host diversification of this parasitoid wasp.

In the previous study on the chromosome-level genome assembly of *B. dioryctriae*, the genome of *B. dioryctriae* was compared with those of 13 other species (*Aphidius gifuensis*, *Apis mellifera*, *Athalia rosae*, *Chouioia cunea*, *Eretmocerus hayati*, *Gonatopus flavifemur*, *Nasonia vitripennis*, *Orussus abietinus*, *Ooceraea biroi*, *Polistes dominula*, *Pteromalus puparum*, *Trichogramma pretiosum*, and *Telenomus remus*), a subset of *BdioTrets* was identified as having undergone significant expansion in *B. dioryctriae*. Fourteen candidate genes for RT-qPCR validation were selected according to four combined objective criteria. First, a full-length coding sequence containing a complete MFS domain and transmembrane-helix architecture. Second, conservation of key functional residues including the GRR/K motif, the TM7 QL/FS motif and the TM10 tryptophan residue. Third, belonging to the expanded gene repertoire. Fourth, showing pronounced tissue-biased expression (log_2_(FPKM + 1) ≥ 5.23 in at least one tissue). Genes fulfilling all four criteria were retained for experimental validation. These candidates included *BdioTret3-6*, *9*, *13*, *24*, *25*, *33*, *35*, *40*, *47*, *49*, and *67*. Notably, *BdioTret40* belongs to the expanded gene set; *BdioTret3* and *6* are classified as *Tret1*, and *BdioTret4* is classified as *Tret1-2*. Multiple sequence alignment revealed that the candidate BdioTRETs universally retain key functional motifs: the GRR/K residues involved in conformational changes and membrane topology, the tryptophan (W) residue in TM10 associated with substrate selectivity, and the QL/FS motif in TM7 related to transport activity. The tryptophan residue in TM10 exhibited the highest conservation level at 85.71%, which was consistent with the conserved signature of the GLUT/SLC2A family [11,12].

### 3.5. Phylogenetic Analysis of Candidate BdioTrets

**Figure 4:**
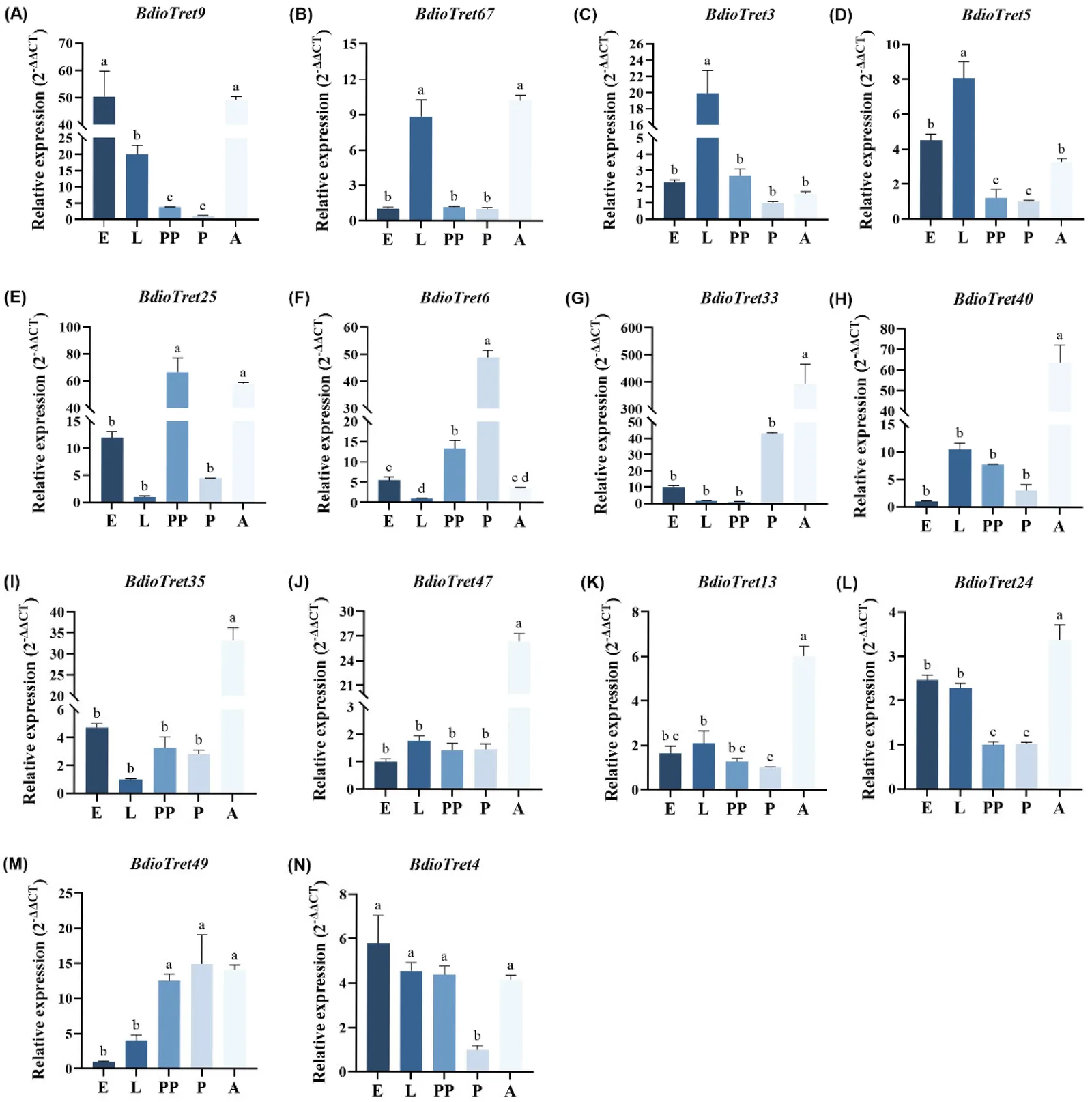
Expression profiles of candidate *BdioTrets* during different developmental stages. E, eggs; L, larvae; PP, prepupae; P, pupae; A, adults. Note: Data are presented as mean ± standard deviation (SD). Three independent biological replicates were performed, and each biological replicate was assayed with three technical RT-qPCR replicates. Technical replicates were averaged prior to statistical analysis. Different lowercase letters above bars indicate significant differences. Panels (**A**)–(**N**) show the relative expression levels of *BdioTret9*, *BdioTret67*, *BdioTret3*, *BdioTret5*, *BdioTret25*, *BdioTret6*, *BdioTret33*, *BdioTret40*, *BdioTret35*, *BdioTret47*, *BdioTret13*, *BdioTret24*, *BdioTret49*, and *BdioTret4*, respectively.

The transcriptomic expression profiles of the 14 candidate *BdioTret* genes exhibited distinct developmental stage-specific expression patterns across the complete life cycle of *B. dioryctriae* (Figure 4). The expression profiles of *BdioTrets* exhibited distinct stage-specific patterns across the developmental timeline of the parasitoid wasp (Figure 4). *BdioTret9* was significantly highly expressed during the egg and adult stages. Genes exhibiting high expression during the larval stage included *BdioTret3*, *5*, and *67*. *BdioTret25* exhibited peak expression at the prepupal (PP) stage, and *BdioTret6* reached maximum expression at the pupal (P) stage; notably, *BdioTret25* also remained highly expressed during the adult stage, with expression levels comparable to those observed in the prepupa. The remaining genes—*BdioTret33*, *40*, *35*, *47*, *13*, and *24*—were all predominantly highly expressed in the adult stage. Additionally, two genes displayed sustained high expression across multiple stages: *BdioTret49* was significantly upregulated starting from the prepupal stage, while *BdioTret4* was consistently highly expressed throughout all stages except the pupa. These results indicate that different *BdioTrets* are expressed in a developmentally specific manner, yet they are universally predominant during the adult stage.

**Figure 5:**
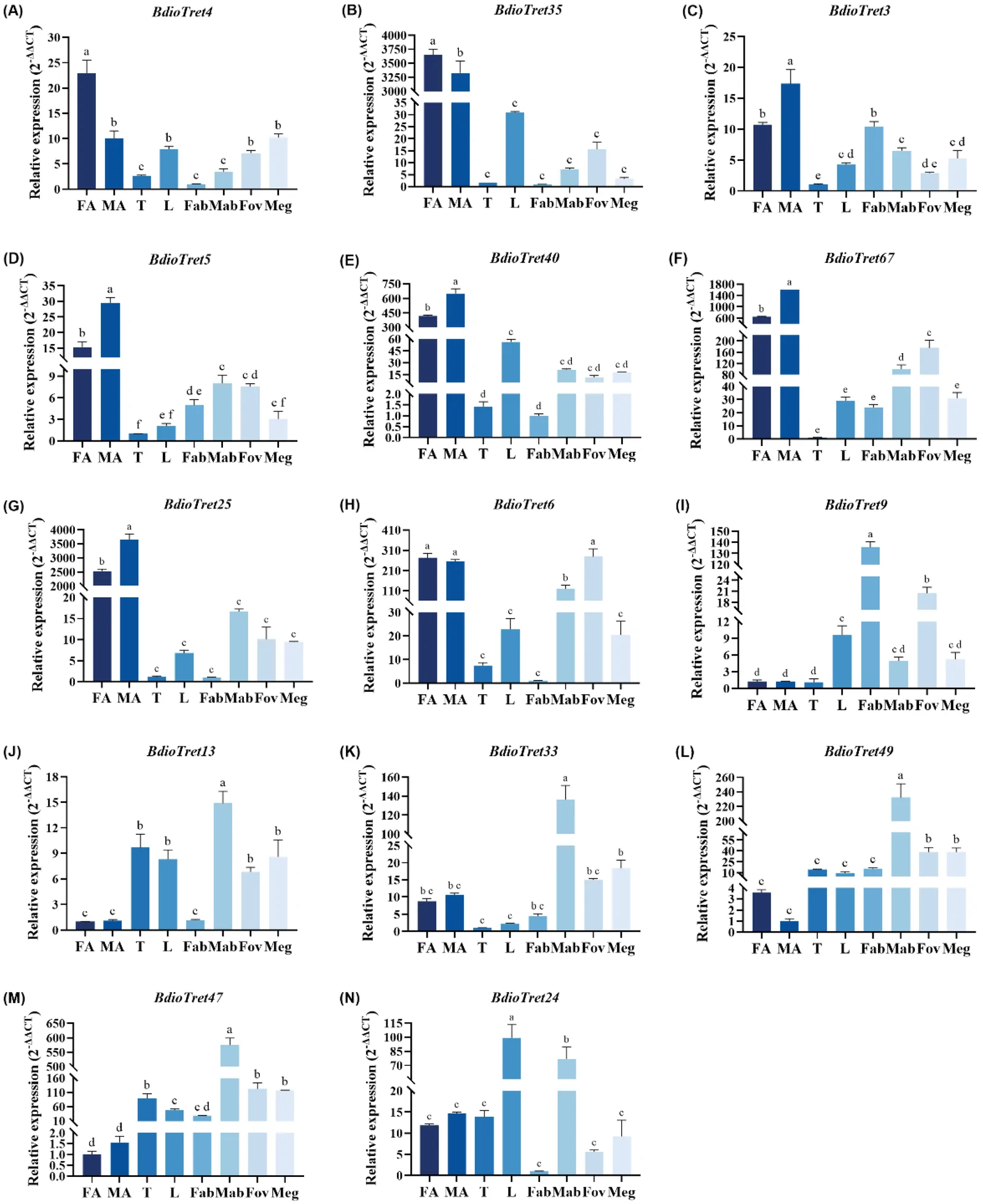
Expression profiles of candidate *BdioTrets* in eight different tissues. FA, female antennae; MA, male antennae; T, male and female thoraxes; L, male and female legs; Fab, female abdomens without ovipositors and digestive tracts; Mab, male abdomens without genitalia and digestive tracts; Fov, female ovipositors; Meg, male genitalia. Note: Data are presented as mean ± standard deviation (SD). Three independent biological replicates were performed, and each biological replicate was assayed with three technical RT-qPCR replicates. Technical replicates were averaged prior to statistical analysis. Different lowercase letters above bars indicate significant differences. Panels (**A**)–(**N**) show the relative expression levels of *BdioTret4*, *BdioTret35*, *BdioTret3*, *BdioTret5*, *BdioTret40*, *BdioTret67*, *BdioTret25*, *BdioTret6*, *BdioTret9*, *BdioTret13*, *BdioTret33*, *BdioTret49*, *BdioTret47*, and *BdioTret24*, respectively.

RT-qPCR validated the expression of 14 candidate genes across eight tissues (Figure 5). For each gene, Pearson correlation was performed between RNA-seq (FPKM) and RT-qPCR (relative expression) values after log_2_(x + 1) transformation (x = FPKM for RNA-seq; x = relative expression for RT-qPCR). Eleven of the 14 *BdioTret* genes (78.57%) exhibited strong positive correlations (Pearson’s correlation coefficient *r* > 0.7, *p* < 0.05), indicating that the gene expression patterns revealed by RNA-seq and RT-qPCR are generally consistent. The detailed Pearson’s correlation coefficient *r* and *p* values are provided in Supplementary Table S4. Among them, *BdioTret4*, *BdioTret24* and *BdioTret49* exhibited *p* values greater than 0.05. Such discrepancies may arise because expression estimates from RNA-seq generally show certain deviations from the actual gene quantification results [37]. In addition, the templates used for subsequent quantitative experiments in this study were not from the same batch as those used for RNA-seq, which may also exert a certain influence on the quantitative results. *BdioTret4*, *35*, *3*, *5*, *40*, *67*, *25*, and *6* were specifically and significantly highly expressed in the antennae, with most exhibiting sexual dimorphism. For instance, BdioTret4 and 35 were significantly highly expressed in the antennae of both sexes, but their expression levels were significantly higher in females than in males. Conversely, *BdioTret3*, *5*, *40*, *67*, and *25* displayed the opposite pattern, with higher expression in males. Several *BdioTrets* were predominantly highly expressed in the abdomen. *BdioTret9* showed the highest expression in the female abdomen, accompanied by a significant secondary peak in the female genitalia. In contrast, *BdioTret13*, *33*, *49*, and *47* peaked in the male abdomen, with significant secondary peaks observed in the genitalia of both sexes. *BdioTret24* exhibited its highest transcript abundance among the tested tissues in mixed-sex legs. Higher expression relative to the male abdomen was preliminarily observed; nevertheless, this finding is to be further validated with sex-separated leg samples in follow-up experiments. In summary, *BdioTrets* display distinct tissue-specific expression patterns in the parasitoid wasp, with predominant high expression in energy-demanding tissues such as the antennae, abdomen, and genitalia. Furthermore, different BdioTRETs exhibit distinct preferences for specific tissue types.

## 4. Discussion

In this study, the genome of *B. dioryctriae* harbored 94 *BdioTrets*, representing the largest Tret family documented in Hymenoptera to date [19]. Phylogenetic analysis corroborated the Blastp results, indicating that the *BdioTrets* family shares the closest relationship with *PcofTrets*, which also belongs to the Chalcidoidea superfamily. This strongly supports the hypothesis that the family has experienced conservative vertical inheritance within the Chalcidoidea, upon which significant lineage-specific expansion has occurred. Such large-scale gene family expansion is not merely a process of simple repetition; rather, it provides abundant genetic raw material for subfunctionalization or neofunctionalization. In insects, this phenomenon is often closely linked to evolutionary innovations in energy homeostasis regulation and environmental adaptation, a pattern relatively common in Lepidopteran insects. For instance, 69 *Trets* were identified in *H. armigera*, where TRET diversity enhances stress response and recovery capabilities [11]. Similarly, in *B. mori* and *S. frugiperda*, large-scale gene duplication of *Trets* has facilitated functional divergence, playing crucial roles in sustaining growth, maintaining metabolic homeostasis, and preserving protein synthesis [19].

The spatiotemporal expression patterns of the candidate *BdioTrets* provide critical evidence for functional divergence within this gene family, aligning closely with the universal importance of insect trehalose metabolism and transport in development and stress responses [19,38]. At the developmental level, the stage-specific expression of different genes forms a highly efficient energy supply system. *BdioTret9* was uniquely expressed during the egg stage, likely meeting the moderate energy demands of hatching, a regulatory mechanism crucial for embryonic development and diapause adaptation as revealed in silkworm transcriptomic analyses [39,40]. The larval stage is critical for rapid growth, supported by the high expression of *BdioTret3*, *5*, and *67*. Upon entering metamorphosis, *BdioTret25* and *BdioTret6* peaked during the prepupal and pupal stages, respectively, suggesting their involvement in energy reserve mobilization for tissue remodeling. Notably, most candidate genes (e.g., *BdioTret9*, *13*, *24*, *33*, *35*, *40*, *47*, *49*) were significantly highly expressed during the adult stage, with some (e.g., *BdioTret4*, *49*) maintaining high expression from the prepupa to the adult. This correlates with the complex physiological activities of adults, mirroring the pattern in *P. xylostella*, where *PxTret1-like* peaks in adults to mediate trehalose transport for coping with drought and cold stress [41].

At the tissue level, the expression specificity of *BdioTrets* further refines functional specialization, reflecting distinct metabolic demands. The antennae exhibited high expression of five genes (*BdioTret4*, *25*, *35*, *40*, *67*), most displaying significant sexual dimorphism. While *BdioTret4* and *35* were highly expressed in the antennae of both sexes, their levels were significantly higher in females; conversely, *BdioTret3*, *5*, *40*, *67*, and *25* exhibited the opposite pattern. This dimorphism likely corresponds to the differing energy requirements for female host location and male mate searching. The elevated transcript abundance of *BdioTret9* in the female abdomen and ovipositor may provide an energy supply for ovipositor function or egg transportation during parasitism. Consistently, trehalose transporters (TRET-family proteins) are documented to mediate trehalose allocation to energy-demanding reproductive tissues in parasitoid wasps, and tissue-specific high expression of *Tret* genes is critical for sustaining female fecundity and reproductive-related physiological activities [15]. Conversely, the expression of *BdioTret13*, *33*, *47*, and *49* in male reproductive tissues likely supports spermatogenesis. Additionally, the specific high expression of *BdioTret24* in the legs (pooled samples of both sexes) corroborates previous findings in *B. mori* regarding muscle-associated energy transport [13]. Given that parasitoid wasp larvae develop inside the host hemocoel, absorbing nutrients directly through their integument [42], this precise tissue-specific regulation enables the wasp to fine-tune trehalose uptake and allocation under different physiological states.

The massive expansion of the *BdioTrets* family in *B. dioryctriae* far exceeds that observed in other insects (typically 2–30 members) [19]. This expansion is hypothesized to be driven by the complexity of the parasitoid life history—such as host recognition and concealed oviposition—necessitating deep subfunctionalization of the *Trets* network. Such expansions are common adaptive strategies, providing enriched genetic resources and enhanced adaptive potential [30]. Indeed, the duplication and diversification of related genes like Trehalase (*Treh*) have conferred evolutionary advantages in Lepidopteran insects [43]. This extensive expansion likely enables *B. dioryctriae* to achieve finer regulation of trehalose transport, thereby better adapting to its complex parasitic life cycle. This aligns with the general trend of expansion observed in metabolism-related gene families within Hymenopteran genomes, reflecting a high degree of evolutionary adaptation to energy metabolism [44]. In conclusion, the *BdioTrets* family constructs a highly flexible energy regulation network through large-scale expansion coupled with refined spatiotemporal control, synergistically supporting the wasp’s life cycle, particularly during the adult stage. While this study provides foundational data and candidate targets for functional validation [29], limitations remain, including sampling under normal conditions and a lack of functional validation. Based on the tissue-specific expression profiles revealed by RT-qPCR, it is hypothesized that *BdioTrets* may participate in trehalose transport to meet the metabolic demands of *B. dioryctriae* during parasitism. The distinct expression patterns across developmental stages further suggest stage-specific functional roles. However, some direct functional validations through heterologous expression and RNA-interference assays are still required in future studies. Future studies should implement stress treatments (e.g., low temperature, drought) [41], conduct genomic collinearity analysis, utilize heterologous expression systems (e.g., *Xenopus laevis* oocytes), and employ RNA interference (RNAi) to silence candidate genes [36]. These endeavors will clarify the molecular mechanisms of expansion and gene function, ultimately advancing our understanding of endoparasitic adaptation while optimizing the genetic improvement of *B. dioryctriae* for enhanced biological control of lepidopteran borers.

## Figures and Tables

**Table 1:** Identification of trehalose transporter genes in *Baryscapus dioryctriae*.

Gene Name	Unigene Length (bp)	Unigene ORF (aa)	Full-Length	TMD (No.)	Classification	BLASTx Best Hit	Query Cover	Ident	ID
*BdioTret1*	3756	1251	Yes	15	uncertain MFS transporter	facilitated trehalose transporter Tret1-like [*Phymastichus coffea*]	59.00%	49.40%	XP_058809364.1
*BdioTret2*	1698	565	Yes	12	confirmed MFS transporter	facilitated trehalose transporter Tret1-2 homolog [*Phymastichus coffea*]	100.00%	78.66%	XP_058797919.1
*BdioTret3*	1635	544	Yes	12	confirmed MFS transporter	facilitated trehalose transporter Tret1 isoform X2 [*Phymastichus coffea*]	90.00%	87.91%	XP_058798228.1
*BdioTret4*	1629	542	Yes	12	confirmed MFS transporter	facilitated trehalose transporter Tret1-like [*Phymastichus coffea*]	100.00%	85.03%	XP_058794243.1
*BdioTret5*	1611	536	Yes	12	confirmed MFS transporter	facilitated trehalose transporter Tret1-like [*Phymastichus coffea*]	100.00%	79.37%	XP_058800267.1
*BdioTret6*	1608	535	Yes	12	confirmed MFS transporter	facilitated trehalose transporter Tret1-like [*Phymastichus coffea*]	100.00%	82.12%	XP_058790847.1
*BdioTret7*	1593	530	Yes	12	confirmed MFS transporter	facilitated trehalose transporter Tret1-like [*Phymastichus coffea*]	91.00%	60.79%	XP_058809015.1
*BdioTret8*	1593	530	Yes	12	confirmed MFS transporter	facilitated trehalose transporter Tret1-like [*Phymastichus coffea*]	91.00%	61.00%	XP_058809015.1
*BdioTret9*	1590	529	Yes	10	confirmed MFS transporter	facilitated trehalose transporter Tret1-like [*Phymastichus coffea*]	95.00%	72.10%	XP_058809364.1
*BdioTret10*	1590	529	Yes	12	confirmed MFS transporter	facilitated trehalose transporter Tret1-like isoform X1 [*Phymastichus coffea*]	97.00%	77.52%	XP_058804247.1
*BdioTret11*	1584	527	Yes	12	confirmed MFS transporter	facilitated trehalose transporter Tret1-like [*Phymastichus coffea*]	88.00%	53.42%	XP_058795122.1
*BdioTret12*	1581	526	Yes	10	confirmed MFS transporter	facilitated trehalose transporter Tret1-like isoform X2 [*Phymastichus coffea*]	90.00%	81.10%	XP_058790660.1
*BdioTret13*	1572	523	Yes	12	confirmed MFS transporter	facilitated trehalose transporter Tret1-like isoform X2 [*Phymastichus coffea*]	100.00%	88.15%	XP_058806691.1
*BdioTret14*	1539	512	Yes	12	confirmed MFS transporter	facilitated trehalose transporter Tret1-like isoform X2 [*Phymastichus coffea*]	99.00%	79.64%	XP_058790853.1
*BdioTret15*	1533	510	Yes	12	confirmed MFS transporter	facilitated trehalose transporter Tret1-like [*Phymastichus coffea*]	93.00%	52.69%	XP_058794789.1
*BdioTret16*	1527	508	Yes	12	confirmed MFS transporter	facilitated trehalose transporter Tret1-like [*Phymastichus coffea*]	93.00%	66.46%	XP_058794789.1
*BdioTret17*	1515	504	Yes	12	confirmed MFS transporter	facilitated trehalose transporter Tret1-like [*Phymastichus coffea*]	94.00%	51.67%	XP_058794789.1
*BdioTret18*	1500	499	Yes	11	confirmed MFS transporter	facilitated trehalose transporter Tret1-like [*Phymastichus coffea*]	95.00%	66.32%	XP_058797261.1
*BdioTret19*	1497	498	Yes	10	confirmed MFS transporter	facilitated trehalose transporter Tret1 [*Nasonia vitripennis*]	92.00%	63.56%	XP_001605654.1
*BdioTret20*	1497	498	Yes	12	confirmed MFS transporter	facilitated trehalose transporter Tret1-like isoform X3 [*Nasonia vitripennis*]	86.00%	76.40%	XP_032454001.1
*BdioTret21*	1491	496	Yes	11	confirmed MFS transporter	facilitated trehalose transporter Tret1-like [*Copidosoma floridanum*]	94.00%	36.42%	XP_014207101.1
*BdioTret22*	1485	494	Yes	11	confirmed MFS transporter	facilitated trehalose transporter Tret1-like [*Phymastichus coffea*]	95.00%	55.96%	XP_058809364.1
*BdioTret23*	1485	494	Yes	12	confirmed MFS transporter	facilitated trehalose transporter Tret1-like [*Phymastichus coffea*]	98.00%	58.68%	XP_058809015.1
*BdioTret24*	1485	494	Yes	12	confirmed MFS transporter	facilitated trehalose transporter Tret1-like [*Phymastichus coffea*]	100.00%	83.00%	XP_058797884.1
*BdioTret25*	1479	492	Yes	11	confirmed MFS transporter	facilitated trehalose transporter Tret1-like [*Phymastichus coffea*]	96.00%	64.27%	XP_058790707.1
*BdioTret26*	1476	491	Yes	12	confirmed MFS transporter	facilitated trehalose transporter Tret1-like isoform X2 [*Phymastichus coffea*]	96.00%	61.31%	XP_058795044.1
*BdioTret27*	1473	490	Yes	12	confirmed MFS transporter	facilitated trehalose transporter Tret1-like isoform X2 [*Phymastichus coffea*]	95.00%	64.73%	XP_058794906.1
*BdioTret28*	1473	490	Yes	10	confirmed MFS transporter	facilitated trehalose transporter Tret1-like [*Phymastichus coffea*]	99.00%	59.50%	XP_058809015.1
*BdioTret29*	1473	490	Yes	10	confirmed MFS transporter	facilitated trehalose transporter Tret1-like [*Phymastichus coffea*]	99.00%	59.50%	XP_058809015.1
*BdioTret30*	1470	489	Yes	12	confirmed MFS transporter	facilitated trehalose transporter Tret1-like [*Phymastichus coffea*]	96.00%	50.74%	XP_058795122.1
*BdioTret31*	1464	487	Yes	11	confirmed MFS transporter	facilitated trehalose transporter Tret1-like [*Phymastichus coffea*]	99.00%	82.82%	XP_058809108.1
*BdioTret32*	1461	486	Yes	12	confirmed MFS transporter	facilitated trehalose transporter Tret1-like [*Phymastichus coffea*]	74.00%	33.97%	XP_058802577.1
*BdioTret33*	1449	482	Yes	12	confirmed MFS transporter	facilitated trehalose transporter Tret1-like [*Phymastichus coffea*]	96.00%	55.08%	XP_058795122.1
*BdioTret34*	1446	481	Yes	12	confirmed MFS transporter	facilitated trehalose transporter Tret1-like isoform X2 [*Phymastichus coffea*]	97.00%	53.96%	XP_058794906.1
*BdioTret35*	1446	481	Yes	12	confirmed MFS transporter	facilitated trehalose transporter Tret1-like isoform X2 [*Phymastichus coffea*]	99.00%	60.21%	XP_058795044.1
*BdioTret36*	1443	480	Yes	11	confirmed MFS transporter	facilitated trehalose transporter Tret1-like isoform X2 [*Phymastichus coffea*]	99.00%	85.47%	XP_058797921.1
*BdioTret37*	1443	480	Yes	10	confirmed MFS transporter	facilitated trehalose transporter Tret1-like [*Phymastichus coffea*]	96.00%	50.75%	XP_058809364.1
*BdioTret38*	1443	480	Yes	10	confirmed MFS transporter	facilitated trehalose transporter Tret1-like [*Copidosoma floridanum*]	93.00%	55.65%	XP_014215782.1
*BdioTret39*	1443	480	Yes	12	confirmed MFS transporter	facilitated trehalose transporter Tret1-like isoform X2 [*Phymastichus coffea*]	98.00%	64.56%	XP_058795044.1
*BdioTret40*	1440	479	Yes	12	confirmed MFS transporter	facilitated trehalose transporter Tret1-like [*Phymastichus coffea*]	98.00%	82.34%	XP_058797205.1
*BdioTret41*	1440	479	Yes	12	confirmed MFS transporter	facilitated trehalose transporter Tret1-like [*Phymastichus coffea*]	100.00%	68.57%	XP_058788829.1
*BdioTret42*	1434	477	Yes	9	partial sequence	facilitated trehalose transporter Tret1-like [*Phymastichus coffea*]	92.00%	48.86%	XP_058789529.1
*BdioTret43*	1434	477	Yes	11	confirmed MFS transporter	facilitated trehalose transporter Tret1-like [*Phymastichus coffea*]	97.00%	45.44%	XP_058809364.1
*BdioTret44*	1434	477	Yes	12	confirmed MFS transporter	facilitated trehalose transporter Tret1 [*Trichogramma pretiosum*]	94.00%	48.10%	XP_014225063.1
*BdioTret45*	1434	477	Yes	12	confirmed MFS transporter	facilitated trehalose transporter Tret1-like isoform X3 [*Nasonia vitripennis*]	90.00%	67.29%	XP_032454001.1
*BdioTret46*	1431	476	Yes	10	confirmed MFS transporter	facilitated trehalose transporter Tret1-like [*Phymastichus coffea*]	70.00%	36.02%	XP_058802577.1
*BdioTret47*	1428	475	Yes	12	confirmed MFS transporter	facilitated trehalose transporter Tret1-like isoform X2 [*Phymastichus coffea*]	98.00%	63.79%	XP_058794906.1
*BdioTret48*	1428	475	Yes	12	confirmed MFS transporter	facilitated trehalose transporter Tret1-like [*Phymastichus coffea*]	99.00%	58.90%	XP_058795122.1
*BdioTret49*	1425	474	Yes	12	confirmed MFS transporter	facilitated trehalose transporter Tret1-like [*Phymastichus coffea*]	99.00%	63.46%	XP_058795122.1
*BdioTret50*	1422	473	Yes	10	confirmed MFS transporter	facilitated trehalose transporter Tret1-like [*Phymastichus coffea*]	97.00%	46.10%	XP_058809364.1
*BdioTret51*	1407	468	Yes	11	confirmed MFS transporter	facilitated trehalose transporter Tret1-like [*Phymastichus coffea*]	98.00%	57.71%	XP_058809015.1
*BdioTret52*	1404	467	Yes	12	confirmed MFS transporter	facilitated trehalose transporter Tret1-like [*Phymastichus coffea*]	100.00%	84.15%	XP_058792109.1
*BdioTret53*	1401	466	Yes	11	confirmed MFS transporter	facilitated trehalose transporter Tret1-like [*Phymastichus coffea*]	95.00%	60.00%	XP_058797205.1
*BdioTret54*	1398	465	Yes	9	partial sequence	facilitated trehalose transporter Tret1-like [*Nasonia vitripennis*]	94.00%	56.06%	XP_001607216.2
*BdioTret55*	1398	465	Yes	9	partial sequence	facilitated trehalose transporter Tret1-like [*Phymastichus coffea*]	97.00%	50.33%	XP_058809364.1
*BdioTret56*	1392	463	Yes	11	confirmed MFS transporter	facilitated trehalose transporter Tret1 [*Trichogramma pretiosum*]	96.00%	48.10%	XP_014225063.1
*BdioTret57*	1392	463	Yes	11	confirmed MFS transporter	facilitated trehalose transporter Tret1-like [*Phymastichus coffea*]	99.00%	52.94%	XP_058807164.1
*BdioTret58*	1392	463	Yes	10	confirmed MFS transporter	facilitated trehalose transporter Tret1-like [*Phymastichus coffea*]	97.00%	70.51%	XP_058791205.1
*BdioTret59*	1386	461	Yes	12	confirmed MFS transporter	facilitated trehalose transporter Tret1-like [*Phymastichus coffea*]	98.00%	65.41%	XP_058804411.1
*BdioTret60*	1386	461	Yes	12	confirmed MFS transporter	facilitated trehalose transporter Tret1-like [*Phymastichus coffea*]	99.00%	55.92%	XP_058789529.1
*BdioTret61*	1383	460	Yes	12	confirmed MFS transporter	facilitated trehalose transporter Tret1-like [*Phymastichus coffea*]	100.00%	88.04%	XP_058804383.1
*BdioTret62*	1383	460	Yes	12	confirmed MFS transporter	facilitated trehalose transporter Tret1-like [*Phymastichus coffea*]	100.00%	88.04%	XP_058804383.1
*BdioTret63*	1380	459	Yes	12	confirmed MFS transporter	facilitated trehalose transporter Tret1-like [*Phymastichus coffea*]	100.00%	79.08%	XP_058809126.1
*BdioTret64*	1380	459	Yes	12	confirmed MFS transporter	facilitated trehalose transporter Tret1-like [*Phymastichus coffea*]	100.00%	69.93%	XP_058807164.1
*BdioTret65*	1380	459	Yes	12	confirmed MFS transporter	facilitated trehalose transporter Tret1-like [*Phymastichus coffea*]	100.00%	80.61%	XP_058809126.1
*BdioTret66*	1377	458	Yes	12	confirmed MFS transporter	facilitated trehalose transporter Tret1-like [*Phymastichus coffea*]	100.00%	71.83%	XP_058807157.1
*BdioTret67*	1374	457	Yes	12	confirmed MFS transporter	facilitated trehalose transporter Tret1-like [*Phymastichus coffea*]	100.00%	61.71%	XP_058797205.1
*BdioTret68*	1374	457	Yes	12	confirmed MFS transporter	facilitated trehalose transporter Tret1-like [*Phymastichus coffea*]	98.00%	73.94%	XP_058807157.1
*BdioTret69*	1374	457	Yes	10	confirmed MFS transporter	facilitated trehalose transporter Tret1-like [*Phymastichus coffea*]	99.00%	47.36%	XP_058807157.1
*BdioTret70*	1368	455	Yes	12	confirmed MFS transporter	facilitated trehalose transporter Tret1-like [*Phymastichus coffea*]	100.00%	70.26%	XP_058791206.1
*BdioTret71*	1368	455	Yes	12	confirmed MFS transporter	facilitated trehalose transporter Tret1-like [*Phymastichus coffea*]	97.00%	51.92%	XP_058807157.1
*BdioTret72*	1365	454	Yes	12	confirmed MFS transporter	facilitated trehalose transporter Tret1-like [*Phymastichus coffea*]	100.00%	75.77%	XP_058804411.1
*BdioTret73*	1365	454	Yes	12	confirmed MFS transporter	facilitated trehalose transporter Tret1-like [*Phymastichus coffea*]	100.00%	75.77%	XP_058804411.1
*BdioTret74*	1347	448	Yes	12	confirmed MFS transporter	facilitated trehalose transporter Tret1-like [*Phymastichus coffea*]	98.00%	71.46%	XP_058800116.1
*BdioTret75*	1344	447	Yes	12	confirmed MFS transporter	facilitated trehalose transporter Tret1 [*Nasonia vitripennis*]	97.00%	68.20%	XP_003425309.1
*BdioTret76*	1314	437	Yes	12	confirmed MFS transporter	facilitated trehalose transporter Tret1-like [*Phymastichus coffea*]	99.00%	59.73%	XP_058791206.1
*BdioTret77*	1311	436	Yes	11	confirmed MFS transporter	facilitated trehalose transporter Tret1-like [*Phymastichus coffea*]	98.00%	53.61%	XP_058807157.1
*BdioTret78*	1305	434	Yes	10	confirmed MFS transporter	facilitated trehalose transporter Tret1-like [*Phymastichus coffea*]	99.00%	61.30%	XP_058791206.1
*BdioTret79*	1296	431	Yes	10	confirmed MFS transporter	facilitated trehalose transporter Tret1 [*Nasonia vitripennis*]	98.00%	53.57%	XP_001604518.1
*BdioTret80*	1275	424	Yes	9	partial sequence	facilitated trehalose transporter Tret1-like [*Phymastichus coffea*]	100.00%	82.15%	XP_058809108.1
*BdioTret81*	1233	410	Yes	10	confirmed MFS transporter	facilitated trehalose transporter Tret1-like [*Phymastichus coffea*]	96.00%	46.08%	XP_058809364.1
*BdioTret82*	1233	410	Yes	10	confirmed MFS transporter	facilitated trehalose transporter Tret1-2 homolog [*Phymastichus coffea*]	96.00%	83.16%	XP_058798089.1
*BdioTret83*	1182	393	No	10	partial sequences	facilitated trehalose transporter Tret1-like [*Phymastichus coffea*]	99.00%	52.20%	XP_058794789.1
*BdioTret84*	1149	382	No	10	partial sequences	facilitated trehalose transporter Tret1-like [*Nasonia vitripennis*]	99.00%	26.75%	XP_001602709.1
*BdioTret85*	1122	373	No	8	partial sequences	facilitated trehalose transporter Tret1-like [*Phymastichus coffea*]	99.00%	53.55%	XP_058809015.1
*BdioTret86*	1110	369	No	9	partial sequences	facilitated trehalose transporter Tret1-like [*Phymastichus coffea*]	97.00%	60.45%	XP_058809015.1
*BdioTret87*	1104	367	No	7	partial sequences	facilitated trehalose transporter Tret1-like [*Phymastichus coffea*]	96.00%	49.16%	XP_058809364.1
*BdioTret88*	1071	356	No	8	partial sequences	facilitated trehalose transporter Tret1-like [*Phymastichus coffea*]	88.00%	46.03%	XP_058788829.1
*BdioTret89*	1047	348	No	9	partial sequences	facilitated trehalose transporter Tret1-like [*Phymastichus coffea*]	99.00%	65.12%	XP_058802577.1
*BdioTret90*	1041	346	No	7	partial sequences	facilitated trehalose transporter Tret1-like [*Phymastichus coffea*]	99.00%	50.51%	XP_058809015.1
*BdioTret91*	963	320	No	7	partial sequences	facilitated trehalose transporter Tret1-like [*Phymastichus coffea*]	75.00%	32.93%	XP_058802577.1
*BdioTret92*	909	302	No	6	partial sequences	facilitated trehalose transporter Tret1-like [*Phymastichus coffea*]	85.00%	34.09%	XP_058802577.1
*BdioTret93*	885	294	No	6	partial sequences	facilitated trehalose transporter Tret1-like [*Copidosoma floridanum*]	98.00%	32.66%	XP_014207101.1
*BdioTret94*	492	163	No	3	partial sequences	PREDICTED: facilitated trehalose transporter Tret1-like [*Ceratosolen solmsi marchali*]	96.00%	48.45%	XP_011498158.1

## Data Availability

The *B. dioryctriae* genome assembly and annotation (including all 94 BdioTret sequences) are available in NCBI under BioProject PRJNA1099397 (GenBank assembly accession: GCA_040869075.1).

## Supplementary Materials

The following supporting information can be downloaded at: https://www.mdpi.com/article/10.3390/insects17101011/s1, Figure S1: Standard curves for RT-qPCR primer efficiency validation of the two reference genes. (A) *BdioRPL18*; (B) *BdioGAPDH*. Ten-fold serial dilutions were prepared from a pooled cDNA sample generated by mixing equal amounts of ten individual cDNA templates, with Ct values plotted against the logarithm of the dilution factor [log_10_ (dilution factor)]. The coefficient of determination (R^2^) and amplification efficiency (E %) are indicated in each panel.; Table S1: Primers for RT-qPCR used in this study. Table S2: TRETs sequence information of seven hymenopteran species used for ML tree. Table S3: Descriptive statistics and percentile distribution of log_2_(FPKM+1) values for 94 BdioTret genes across eight examined tissues. Sample size = 752, mean = 1.51. Table S4: Pearson’s correlation analysis between RNA-seq and RT-qPCR for the 14 candidate BdioTret genes across eight examined tissues.
