# Supplementary material for: Screening and Expression Characteristics of Trehalose Transporter Genes in the Parasitic Wasp Baryscapus dioryctriae (Hymenoptera: Eulophidae)
Source: Insects. 2026 Sep 30;17(10):1011. doi: 10.3390/insects17101011 (PMC13705112; doi:10.3390/insects17101011)
Supplement: Supplementary file 1 [file insects-17-01011-s001.zip › insects-4522033-supplementary-Figure S1.pdf]

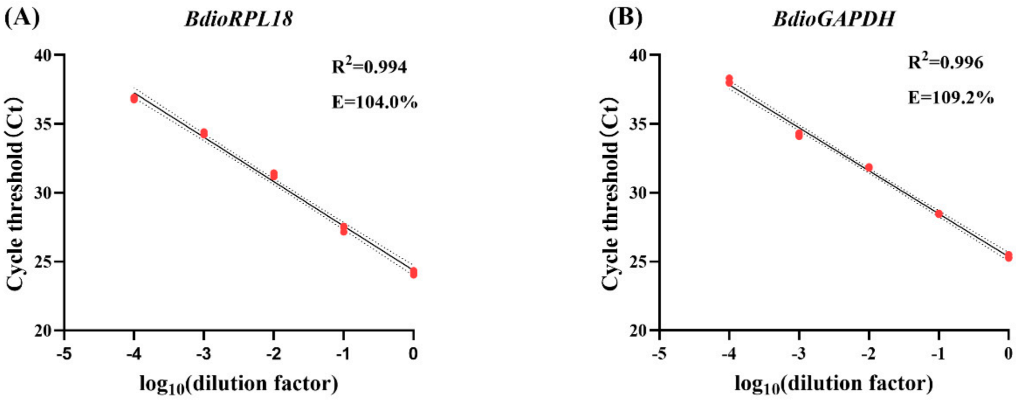

Figure S1 Standard curves for RT-qPCR primer efficiency validation of the two reference genes. (A) *BdioRPL18*; (B) *BdioGAPDH*. Ten-fold serial dilutions were prepared from a pooled cDNA sample generated by mixing equal amounts of ten individual cDNA templates, with Ct values plotted against the logarithm of the dilution factor [$\log_{10}$ (dilution factor)]. The coefficient of determination (R^2) and amplification efficiency (E %) are indicated in each panel.
